# Supplementary material for: The Effects of Temperature Management on Brain Microcirculation, Oxygenation and Metabolism
Source: Brain Sci. 2022 Oct 21;12(10):1422. doi: 10.3390/brainsci12101422 (PMC9599843; doi:10.3390/brainsci12101422)
Supplement: Supplementary file 1 [file brainsci-12-01422-s001.zip › brainsci-1870865 - Supplementary.pdf]

brain sciences

Supplementary Materials

The Effects of Temperature Management on Brain Microcirculation, Oxygenation and Metabolism

Katia Donadello [1,2,*], Fuhong Su [1], Filippo Annoni [1], Sabino Scolletta [1,3], Xinrong He [1,4], Lorenzo Peluso [1], Leonardo Gottin [5], Enrico Polati [2], Jacques Creteur [1], Olivier De Witte [6], Jean-Louis Vincent [1], Daniel De Backer [1,7] and Fabio Silvio Taccone [1]

1 Department of Intensive Care, Erasme Hospital, Free University of Brussels, Route de Lennik 808, 1070 Brussels, Belgium
2 Department of Anesthesia and Intensive Care B, Department of Surgery, Dentistry, Gynaecology and Paediatrics, University of Verona, AOUI-University Hospital Integrated Trust of Verona, Policlinico G.B. Rossi, Piazzale Ludovico Scuro, 37134 Verona, Italy
3 Service of Intensive and Critical Care Medicine, Department of Medical Science, Surgery and Neuroscience, University of Siena, 53100 Siena, Italy
4 Department of Intensive Care Medicine, Sun Yat-sen University Cancer Center, Guangzhou 510060, China
5 Departement of Cardio-Thoracic Anesthesia and Intensive Care, Department of Surgery, Dentistry, Gynaecology and Paediatrics, University of Verona, AOUI-University Hospital Integrated Trust of Verona, Piazzale Aristide Stefani, 37100 Verona, Italy
6 Department of Neurosurgery, Erasme Hospital, Free University of Brussels, Route de Lennik 808, 1070 Brussels, Belgium
7 Department of Intensive Care, CHIREC, 1420 Braine L'Alleud, Belgium
* Correspondence: katia.donadello@univr.it

Citation: Donadello, K.; Su, F.; Annoni, F.; Scolletta, S.; He, X.; Peluso, L.; Gottin, L.; Polati, E.; Creteur, J.; De Witte, O.; et al. The Effects of Temperature Management on Brain Microcirculation, Oxygenation and Metabolism. *Brain Sci.* **2022**, *12*, 1422. https://doi.org/10.3390/brainsci12101422

Academic Editors: Claudio Torres andAlexander Gourine

Received: 1 August 2022
Accepted: 20 October 2022
Published: 21 October 2022

Publisher's Note: MDPI stays neutral with regard to jurisdictional claims in published maps and institutional affiliations.

Copyright: © 2022 by the author. Licensee MDPI, Basel, Switzerland. This article is an open access article distributed under the terms and conditions of the Creative Commons Attribution (CC BY) license (https://creativecommons.org/licenses/by/4.0/).

Abstract: *Purpose*: Target temperature management (TTM) is often used in patients after cardiac arrest, but the effects of cooling on cerebral microcirculation, oxygenation and metabolism are poorly understood. We studied the time-course of these variables in a healthy swine model. *Methods*: Fifteen invasively monitored, mechanically ventilated pigs were allocated to sham procedure (normothermia, NT; n = 5), cooling (hypothermia, HT, n = 5) or cooling with controlled oxygenation (HT-Oxy, n = 5). Cooling was induced by cold intravenous saline infusion, ice packs and nasal cooling to achieve a body temperature of 33-35°C. After 6 hours, animals were rewarmed to baseline temperature (within 5 hours). The microvascular cerebral network was evaluated (at baseline and 2, 7 and 12 hours thereafter) using sidestream dark-field (SDF) video-microscopy. Cerebral blood flow (laser Doppler MNP100XP, Oxyflow, Oxford Optronix, Oxford, UK), oxygenation ($PbtO_2$, Licox catheter, Integra Lifesciences, USA) and lactate/pyruvate ratio (LPR) using brain microdialysis (CMA, Stockholm, Sweden) were measured hourly. *Results*: In HT animals, cerebral functional capillary density (FCD) and proportion of small-perfused vessels (PSPV) significantly decreased over time during cooling phase; concomitantly, $PbtO_2$ increased and LPR decreased. After rewarming, all microcirculatory variables returned towards normal values, except LPR which increased during the rewarming phase in the two groups subjected to HT when compared to the group maintained at normothermia. *Conclusions*: In healthy animals, TTM cooling at 33-35°C can be associated with alterations in the cerebral microcirculation during cooling and altered metabolism at rewarming.

Keywords: microvascular flow; cerebral oxygenation; hypothermia; brain metabolism; lactate

Table S1. Evolution of systemic hemodynamics over time in animals treated with normothermia (NT, n = 5), with hypothermia (HT, n = 5) and with hypothermia associated with constant oxygenation (HT-Oxy, n = 5). Data are presented as mean ± SD. HR = heart rate; MAP = mean arterial pressure; MPAP = mean pulmonary arterial pressure; PAOP = pulmonary arterial occlusive pressure; RAP = right atrial pressure; SVRI = systemic vascular resistance index; PVRI = pulmonary vascular resistance index; DO_2 = oxygen delivery; VO_2 = oxygen consumption; OER = oxygen extraction rate. In *post hoc* multiple group comparison, significant differences among groups are indicated, whenever necessary (**NT = reference; [a] = vs. HT group; [b] = vs. HT-Oxy group**).

	Group	Baseline	2 Hours	7 Hours	12 Hours	ANOVA Interaction Time-Group
**Body Temperature, °C**	NT	39.2 ± 0.6	39.5 ± 0.8[a,b]	40.2 ± 0.4[a,b]	40.1 ± 0.5 [a,b]	$p < 0.001$
	HT	38.8 ± 0.7	34.9 ±0.9	34.2 ± 0.7	38.7 ± 0.5	
	HT-Oxy	38.4 ± 0.6	34.1 ± 0.5	34.3 ± 0.5	38.6 ± 0.2	
**Brain Temperature, °C**	NT	38.2 ± 0.7	38.1 ± 0.9[a,b]	38.7 ± 0.8[a,b]	38.9 ± 0.9	$p < 0.001$
	HT	37.7 ± 0.5	32.1 ± 0.5	33.1 ± 1.4	37.6 ± 0.7	
	HT-Oxy	37.9 ± 0.7	32.4 ± 1.3	33.9 ± 0.7	38.1 ± 0.5	
**HR, beats/min**	NT	75 ± 11	79 ± 13	82 ±10[a,b]	78 ± 9	$p < 0.001$
	HT	69 ± 5	66 ± 5	55 ± 4	79 ± 13	
	HT-Oxy	73 ± 8	63 ± 9	57 ± 4	74 ± 8	
**Cardiac Index, L/min.m²**	NT	3.7 ± 0.3	4.1 ± 0.3	3.9 ± 0.8[a]	4.2 ± 0.3	$p < 0.001$
	HT	3.7 ± 0.4	3.8 ± 0.6	2.7 ± 0.3	3.9 ± 0.8	
	HT-Oxy	4.1 ± 0.4	4.2 ± 0.6	3.3 ± 0.3	4.6 ± 0.5	
**Stroke Volume, mL**	NT	47 ± 6	50 ± 6	48 ± 5	50 ± 4	$p = 0.33$
	HT	54 ± 6	57 ± 6	49 ± 9	49 ± 10	
	HT-Oxy	53 ± 9	59 ± 10	51 ± 8	57 ± 8	
**MAP, mmHg**	NT	110 ± 7	110 ± 9	92 ±12	98 ± 10	$p = 0.39$
	HT	108 ± 9	105 ± 8	86 ± 7	88 ± 5	
	HT-Oxy	107 ± 7	103 ± 8	86 ± 6	82 ± 6	
**PAOP, mmHg**	NT	10 ± 4	11 ± 4	11 ± 4	11 ± 3	$p < 0.001$
	HT	10 ± 3	12 ± 4	11±	11 ± 3	
	HT-Oxy	9 ± 2	11 ± 2	8 ± 2	10 ± 2	
**RAP, mmHg**	NT	6 ± 4	7 ± 5	8 ± 4	7 ± 4	$p = 0.60$
	HT	5 ± 4	7 ± 5	7 ± 5	6 ± 4	
	HT-Oxy	6 ± 3	8 ± 2	6 ± 3	7 ± 3	
**SVRI, dynes.sec.cm⁻⁵**	NT	1958 ± 454	1691 ± 929	1790 ± 442[a]	1791 ± 276[b]	$p = 0.006$
	HT	2225 ± 269	2182 ± 482	2600 ± 405	1727 ± 357	
	HT-Oxy	1835 ± 267	1778 ± 353	1929 ± 318[a]	1262 ± 226	
**PVRI, dynes.sec.cm⁻⁵**	NT	241 ± 54	263 ± 23	251 ± 66[a]	252 ± 44	$p < 0.001$
	HT	318 ± 55	338 ± 64	419 ± 95	333 ± 89	
	HT-Oxy	300 ± 91	282 ± 99	381 ± 107	272 ± 19	
**DO_2, mL**	NT	502 ± 95	536 ± 86	520 ± 225	603 ± 202	$p = 0.02$
	HT	481 ± 53	514 ± 52	406 ± 54	547 ± 99	
	HT-Oxy	478 ± 44	498 ± 119	411 ± 58	553 ± 656	
**VO_2, mL**	NT	231 ± 42	226.89 ± 41.12[a]	206.55 ± 56.69	222.98 ± 42.15	$p < 0.001$

	HT	215 ± 28	144.96 ±43.85	151.47 ± 31.88	228.20 ± 28.97	
	HT-Oxy	225 ± 21	206.43 ± 41.81	161.79 ± 34.82	262.37 ± 53.54	
**OER, %**	NT	48 ± 2	48 ± 6[a]	45 ± 8	46 ± 6	$p < 0.001$
	HT	45 ± 4	29 ± 11	38 ± 8	42 ± 3	
	HT-Oxy	47 ± 3.8	42 ± 3	39 ± 5	47 ± 5	
**SvO_2, %**	NT	55 ± 4	56 ± 5	54 ± 4	55 ± 4	$p < 0.001$
	HT	57 ± 4	71 ± 6	66 ± 7	59 ± 2	
	HT-Oxy	55 ± 3	60 ± 2	65 ± 4	54 ± 5	
**Lactate, mEq/L**	NT	1 ± 0.2	0.7 ± 0.2	0.7 ± 0.0	0.8 ± 0.2	$p = 0.65$
	HT	0.9 ± 0.3	0.6 ± 0.2	0.6 ± 0.1	0.6 ± 0.1	
	HT-Oxy	1.1 ± 0.3	0.9 ± 0.4	0.7 ± 0.2	0.6 ± 0.1	

Table S2. Evolution of systemic respiratory variables over time in animals treated with normothermia (NT, n = 5), with hypothermia (HT, n = 5) and with hypothermia associated with constant oxygenation (HT-Oxy, n = 5). Data are presented as mean ± SD. In *post hoc* multiple group comparison, significant differences among groups are indicated, whenever necessary (**NT = reference; [a] = vs. HT group; [b] = vs. HT-Oxy group**).

	Group	Baseline	2 Hours	7 Hours	12 Hours	ANOVA Interaction Time-Group
**SaO_2, %**	NT	99 ± 1	99 ± 1	99 ± 1	99 ± 1	$p = 0.30$
	HT	99 ± 0	99 ± 0	99 ± 0	98 ± 0	
	HT-Oxy	99 ± 0	99 ± 1	99 ± 1	99 ± 0	
**PaO_2/FiO_2**	NT	455 ± 30	429 ± 41[a]	409 ± 43[a,b]	393 ± 51[a]	$p < 0.001$
	HT	455 ± 13	523 ± 27	586 ± 15	489 ± 21	
	HT-Oxy	436 ± 26	591 ± 35	600 ± 20	431 ± 29	
**PaO_2, mmHg**	NT	184 ±11	174 ± 17[a]	162 ± 15[a]	162 ± 26[a]	$p < 0.001$
	HT	183 ± 7	210 ± 13[b]	234 ± 10[b]	195 ± 9	
	HT-Oxy	177 ± 6	176 ± 4	170 ± 3	174 ± 11	
**pH**	NT	7.51 ± 0.03	7.49 ± 0.02	7.49 ± 0.02	7.47 ± 0.04[a,b]	$p = 0.17$
	HT	7.47 ± 0.04	7.47 ± 0.02	7.47 ± 0.04	7.41 ± 0.01	
	HT-Oxy	7.46 ± 0.03	7.45 ± 0.05	7.44 ± 0.03	7.40 ± 0.03	
**$PaCO_2$, mmHg**	NT	41 ± 3	41 ± 4	39 ± 3	35 ± 2[b]	$p < 0.001$
	HT	37 ± 4	38 ± 3	38 ± 2	41 ± 2	
	HT-Oxy	41 ± 4	39 ± 3	39 ± 3	43 ± 1	

Table S3. Evolution of biological variables over time in animals treated with normothermia (NT, n = 5), with hypothermia (HT, n = 5) and with hypothermia associated with constant oxygenation (HT-Oxy, n = 5). Data are presented as mean ± SD. In *post hoc* multiple group comparison, significant differences among groups are indicated, whenever necessary (**NT = reference; [a] = vs. HT group; [b] = vs. HT-Oxy group**).

	Group	Baseline	2 Hours	7 Hours	12 Hours	ANOVA Interaction Time-Group
**Fluid amount, mL**	NT	2650 ± 518	3550 ± 716	5150 ± 1342	6150 ± 285[a,b]	$p = 0.0047$
	HT	2160 ± 1033	3460 ± 890	5630 ± 1171	7280 ± 333	
	HT-Oxy	2850 ± 224	4900 ± 518	6800 ± 411	7300 ± 209	
**Urine output, mL**	NT	414 ± 51	727 ± 166	1726 ± 343	2686 ± 524	$p = 0.0062$
	HT	320 ± 57	787 ± 349	2167 ± 685	2808 ± 828	

	HT-Oxy	424 ± 199	1456 ± 383	2708 ± 576	3186 ± 267	
**Lactate, mEq/L**	NT	1 ± 0.2	0.7 ± 0.2	0.7 ± 0.0	0.8 ± 0.2	$p = 0.6541$
	HT	0.9 ± 0.3	0.6 ± 0.2	0.6 ± 0.1	0.6 ± 0.1	
	HT-Oxy	1.1 ± 0.3	0.9 ± 0.4	0.7 ± 0.2	0.6 ± 0.1	
**Hemoglobin, g/dL**	NT	8.9 ± 1.5	8.8 ± 1.1	8.9 ± 1.8	9.2 ± 1.9	$p = 0.0049$
	HT	9.3 ± 0.6	9.6 ± 1.1	10.8 ± 0.4[b]	10.2 ± 0.7	
	HT-Oxy	8.4 ± 0.1	8.4 ± 0.9	9.0 ± 0.6	8.7 ± 0.6	
**Glucose, mg/dL**	NT	107 ± 14	94 ± 16	80 ± 18	75 ± 10	$p = 0.2630$
	HT	107 ± 18	86 ± 33	88 ± 16	86 ± 12	
	HT-Oxy	103 ± 17	93 ± 17	92 ± 9	74 ± 5	

Table S4. Evolution of cerebral microcirculation over time in animals treated with normothermia (n = 5), with hypothermia (n = 5) and with hypothermia associated with constant oxygenation (n = 5). Data are presented as mean ± SD. FCD = Functional Capillary Density; PSPV = Proportion of Small Perfused Vessels; MFI = Mean Flow Index; HI = Heterogeneity Index; BLD= Brain Laser Doppler. In *post hoc* multiple group comparison, significant differences among groups are indicated, whenever necessary (**NT = reference; [a] = vs. HT group; [b] = vs. HT-Oxy group**).

	**Group**	**Baseline**	**2 Hours**	**7 Hours**	**12 Hours**	**ANOVA Interaction Time-Groups**
**FCD, n/mm**	NT	3.70 ±0.51	3.60 ± 0.56	3.20 ± 0.41[a,b]	3.51 ± 0.31	$p < 0.001$
	HT	3.35 ± 0.39	3.05 ± 0.66	1.73 ± 0.60	2.59 ± 0.22[b]	
	HT-Oxy	4.34 ± 0.70	2.76 ± 0.95	2.14 ± 0.34	4.32 ± 0.25	
**PSPV, %**	NT	86.01 ± 3.73	83.37 ±7.44[a,b]	77.95 ± 10.09[a,b]	75.47 ± 8.57[a]	$p < 0.001$
	HT	79.01 ± 5.71	66.24 ± 12.75	40.83 ± 8.29	58.02 ±6.31[b]	
	HT-Oxy	78.54 ± 3.88	61.92 ± 5.79	53.09 ± 5.25	76.77 ± 1.26	
**MFI**	NT	2.79 ± 0.13	2.8 ± 0.15[a,b]	2.79 ± 0.14[a,b]	2.73 ± 0.16[a]	$p < 0.001$
	HT	2.78 ± 0.14	2.3 ± 0.44	1.79 ± 0.1	2.2 ± 0.07	
	HT-Oxy	2.73 ± 0.09	2.1 ± 0.2	1.75 ± 0.07	2.5 ± 0.14	
**HI PSPV, %**	NT	11.87 ± 5.39	17.49 ± 8.47	20.95 ± 19.19	15.45 ± 7.09	$p = 0.18$
	HT	17.94 ± 9.37	44.09 ± 25.98	60.89 ± 29.32	34.81 ± 16.56	
	HT-Oxy	12.29 ± 6.09	21.87 ± 10.91	32.38 ± 14.71	15.06 ± 1.65	
**HI FCD, %**	NT	39.76 ± 25.29	39.28 ± 15.79	46.49 ± 21.89	32.43 ± 10.56	$p = 0.24$
	HT	36.98 ± 15.52	47.08 ± 33.17	98.63 ± 38.59	42.83 ± 19.62	
	HT-Oxy	41.21 ± 19.81	43.94 ± 22.78	65.30 ± 32.61	45.45 ± 16.77	
**HI MFI, %**	NT	9.09 ± 6.32	12.83 ± 8.36	16.19 ± 7.76[a]	13.32 ± 12.82	$p = 0.24$
	HT	10.79 ± 3.97	22.43 ± 14.14	36.21 ± 6.86	27.25 ± 13.09	
	HT-Oxy	12.80 ± 4.92	16.83 ± 6.87	27.25 ± 13.09	15.97 ± 5.36	
**BLD, %/Units**	NT	100 ± 0	102.51 ± 20.59[a,b]	86.37 ± 12.39[b]	98.62 ± 6.70	$p < 0.001$
	HT	100 ± 0	71.85 ± 17.04	61.21 ± 13.05	92.99 ± 8.95	
	HT-Oxy	100 ± 0	60.72 ± 13.28	53.22 ± 12.15	89.40 ± 5.17	

Table S5. Evolution of cerebral oxygenation and metabolism over time in animals treated with normothermia (n = 5), with hypothermia (n = 5) and with hypothermia associated with constant oxygenation (n = 5). Data are presented as mean ± SD. $PbtO_2$ = Brain Oxygen Tension; cGlu = Cerebral Glucose; cLac = Cerebral Lactate; cGly = Cerebral Glycerol; cGlt = Cerebral Glutamate; LPR = Lactate/Pyruvate Ratio. In *post hoc* multiple group comparison, significant differences among groups are indicated, whenever necessary (**NT = reference; [a] = vs. HT group; [b] = vs. HT-Oxy group**).

	**Group**	**Baseline**	**2 hours**	**7 hours**	**12 hours**	**ANOVA Interaction Time-Groups**
**$PbtO_2$, mmHg**	NT	85± 2	84 ± 3	85 ± 3[a]	85 ± 3	$p < 0.001$
	HT	87 ± 7	91 ± 6	97 ± 6[b]	88 ± 9	
	HT-Oxy	85 ± 4	86 ± 4	86 ± 4	85 ± 4	
**cGlu, mMol/L**	NT	2.96 ± 1.29	2.48 ± 1.63	1.52 ± 0.99	1.58 ± 0.88	$p = 0.97$
	HT	2.80 ± 0.94	2.82 ± 1.09	1.82 ± 1.10	1.62 ± 1.33	
	HT-Oxy	3.06 ± 1.51	2.78 ± 1.28	1.44 ± 1.23	1.46 ± 1.13	
**cLac, mMol/L**	NT	4.84 ±1.68	4.02 ± 1.03	4.94 ± 1.31	3.98 ± 1.42	$p = 0.004$
	HT	3.94 ± 1.84	3.08 ± 1.45	4.03 ± 0.97	4.54 ± 1.22	
	HT-Oxy	3.56 ± 1.43	3.11 ± 1.59	2.86 ± 1.15	4.52 ± 1.11	
**cPyr, μMol/L**	NT	197.8 ± 64.2	215.4 ± 62.8	222.1 ± 50.1	125.4 ± 81.5	$p = 0.76$
	HT	201.2 ± 40.8	189.2 ± 22.6	200.4 ± 46.2	153.2 ± 67.6	
	HT-Oxy	164.0 ± 52.8	144.8 ± 51	137.6 ± 44.2	151.4 ± 16.9	
**cGly, μMol/L**	NT	72.6 ±18.82	70 ± 23.07	43 ± 30.88	27.2 ± 18.03	$p = 0.21$
	HT	65.8 ± 15.93	60.6 ± 17.81	45.8 ± 9.37	39.4 ± 14.43	
	HT-Oxy	66.6 ± 42.45	49.2 ± 37.44	26.2 ± 21.91	28.2 ± 19.83	
**cGlt, μMol/L**	NT	82 ± 54	74± 56	51 ± 49	59 ± 47	$p = 0.57$
	HT	66 ± 19	57 ± 21	55 ± 30	40 ± 12	
	HT-Oxy	63 ± 33	39 ± 27	42 ± 26	49 ± 29	
**LPR**	NT	19.8 ±4.0	20.1 ± 6.6	24.9 ± 8.8	25.7 ± 6.8	$p = 0.03$
	HT	18.9 ± 6.1	17.0 ± 10.1	18.5 ± 6.7	34.3 ± 13.9	
	HT-Oxy	19.6 ± 4.5	19.5 ± 5.2	18.6 ± 7.1	30.3 ± 13.2	
